# Enhanced prediction of hemolytic activity in antimicrobial peptides using deep learning-based sequence analysis

**DOI:** 10.1186/s12859-024-05983-4

**Published:** 2024-11-27

**Authors:** Ibrahim Abdelbaky, Mohamed Elhakeem, Hilal Tayara, Elsayed Badr, Mustafa Abdul Salam

**Affiliations:** 1 Artificial Intelligence Department, Faculty of Computers and Artificial Intelligence, Benha University, Benha, Egypt; 2 School of International Engineering and Science, Jeonbuk National University, Jeonju, 54896 South Korea; 3 Scientific Computing Department, Faculty of Computers and Artificial Intelligence, Benha University, Benha, Egypt; 4 The Egyptian School of Data Science (ESDS), Benha, Egypt; 5 Department of Information Systems, College of Information Technology, Misr University for Science and Technology, Giza, Egypt; 6 Department of Computer Engineering and Information, College of Engineering, Wadi Ad Dwaser, Prince Sattam Bin Abdulaziz University, Al-Kharj, 16273 Saudi Arabia

**Keywords:** Antimicrobial peptide, Deep learning, Hemolytic activity, Therapeutic peptides

## Abstract

Antimicrobial peptides (AMPs) are a promising class of antimicrobial drugs due to their broad-spectrum activity against microorganisms. However, their clinical application is limited by their potential to cause hemolysis, the destruction of red blood cells. To address this issue, we propose a deep learning model based on convolutional neural networks (CNNs) for predicting the hemolytic activity of AMPs. Peptide sequences are represented using one-hot encoding, and the CNN architecture consists of multiple convolutional and fully connected layers. The model was trained on six different datasets: HemoPI-1, HemoPI-2, HemoPI-3, RNN-Hem, Hlppredfuse, and AMP-Combined, achieving Matthew’s correlation coefficients of 0.9274, 0.5614, 0.6051, 0.6142, 0.8799, and 0.7484, respectively. Our model outperforms previously reported methods and can facilitate the development of novel AMPs with reduced hemolytic activity, which is crucial for their therapeutic use in treating bacterial infections.

## Introduction

Peptides are short amino acid sequences bound together by peptide bonds. They are one of the fundamental building blocks of proteins, which are essential molecules for the structure, function, and regulation of living organisms [1]. Peptides can have a wide range of biological activities and functions, including acting as hormones [2], enzymes, signal molecules, and antimicrobial agents [3]. Antimicrobial peptides (AMPs) are a class of peptides that have potent antimicrobial activity against a broad range of microorganisms, including bacteria, fungi, viruses, and parasites [4]. They are produced by various organisms as part of their innate immune response to protect against infections. AMPs are typically small, cationic, and amphipathic, meaning they have both hydrophobic and hydrophilic regions in their structure. These properties allow them to interact with and disrupt the membrane of microbial cells, leading to their death. AMPs are considered a promising alternative to traditional antibiotics [5], which have become less effective because of the advent of bacteria resistant to antibiotics. However, the clinical use of AMPs has been limited by their potential to cause hemolysis [6]. Hemolysis is the destruction of red blood cells, which can be caused by various factors, including exposure to certain drugs, toxins, or chemicals, as well as by physical damage or disease. In the case of AMPs, hemolysis can occur due to their interaction with the membrane of red blood cells, which is similar in structure to that of microbial cells. AMPs can therefore interact with and damage the membrane of red blood cells, leading to their destruction. This can lead to various adverse effects, such as anemia, renal failure, and shock. In addition, it can limit the clinical use of AMPs as antimicrobial agents. Therefore, accurately predicting the hemolytic activity of AMPs is crucial for their safe and effective use [7].

Several computational methods have been proposed for predicting the hemolytic activity of AMPs. One common approach is to use machine learning algorithms to learn a mapping between the sequence and hemolytic activity of AMPs. For example, Support vector machines (SVMs) and Nearest Neighbors were used by Chaudhary et al. [8] to predict the hemolytic activity of AMPs based on peptide characteristics such as residue-based compositions and binary profiles. Similarly, Win, Thet Su et al. [9] proposed a method based on random forest (RF), decision tree (DT), and SVM, to predict the hemolytic activity of AMPs using features underlying the hemolytic activity of peptides based on amino acid composition (AAC), dipeptide composition (DPC), and physicochemical properties (PCP) derived from the AAindex database [10]. For more details about machine learning algorithms for predicting the hemolytic activity [11–15]. More recently, deep learning methods have been applied to the prediction of hemolytic activity of AMPs. For example, Capecchi et al. [16] used a deep learning approach based on a Recurrent Neural Network (RNN) to predict the hemolytic activity of AMPs. Similarly, Salem et al. [17] Proposed a method based on transfer learning to predict the hemolytic activity of AMPs using their amino acid sequences. For more details about deep learning algorithms for predicting the hemolytic activity [18, 19]. These methods, however, have some drawbacks. Some of these methods, for example, rely on manually created features that may not adequately represent the information required for an accurate estimation of hemolytic activity. Additionally, due to overfitting or a lack of diversity in the training data, some of these methods might not generalize well to new and unexplored data. Our study aligns with previous works that apply CNN architectures with one-hot encoding for bioactive peptide identification, such as those by [20–23]. These studies highlight the effectiveness of CNNs for extracting sequence-based features and further support our model’s design choices for predicting hemolytic activity.

To address the limitations of existing approaches, we propose a novel method for predicting the hemolytic activity of antimicrobial peptides (AMPs) using one-hot encoding and deep learning. Our approach mitigates the shortcomings of previous methods by encoding the amino acid sequences of AMPs into a binary matrix, where each row represents an amino acid, and each column corresponds to a specific position within the sequence. While one-hot encoding represents patterns of peptide sequences, CNNs are effective for identifying local features rather than capturing long-range sequential dependencies. Other architectures like RNNs and Transformers are better suited for tasks requiring sequence-based data analysis. Deep learning, with its capacity to automatically learn and extract pertinent features from complex data, is particularly suited to this task. By leveraging deep learning, our method can identify and utilize intricate patterns within the one-hot encoded sequences, thereby enhancing the accuracy of hemolytic activity predictions. We rigorously evaluate our approach using a comprehensive dataset of AMPs with known hemolytic activity levels, benchmarking its performance against several state-of-the-art methods. The experimental results suggest that our proposed method offers a modest improvement over existing techniques. Although these gains are modest, they indicate a robust and reliable approach across multiple datasets.

The remaining sections of this paper are structured as follows: The description of the proposed method is introduced in Sect. Materials and Methods. In Sect. Results and Discussion, the experimental outcomes of the proposed method on various datasets are analyzed. Section Conclusion contains the conclusion.

## Materials and methods

In this section, we introduce the datasets utilized and elaborate on our proposed method for predicting the hemolytic activity of antimicrobial peptides (AMPs) using one-hot encoding representation and deep learning techniques. The combination of diverse datasets and advanced encoding methods underpins the robustness and accuracy of our predictive model.

### Dataset

In this study, we utilized six datasets to train and test the proposed model for predicting hemolytic activity. These datasets include HemoPI-1, HemoPI-2, HemoPI-3 [8], RNN-Hem [16], Hlppredfuse [24], and AMP-Combined [17]. Each dataset originates from different sources such as literature, databases, and experiments, varying in size, composition, and labeling. Some datasets distinguish solely between hemolytic and non-hemolytic peptides, while others further classify hemolytic peptides based on activity levels. The use of multiple datasets is essential due to the lack of consensus on the most reliable dataset for hemolytic activity prediction. Each dataset possesses unique advantages and limitations, such as data quality, diversity, balance, and representation. Therefore, employing different datasets allows for a comprehensive evaluation of the proposed model’s performance and robustness under various conditions. Table 1 summarizes these datasets. Each dataset was divided into training and testing sets in an 80:20 ratio.

Chaudhary et al. [8] developed a dataset for predicting hemolytic peptides, using it to train and evaluate their method, HemoPI. Win et al. [9] applied their method, HemoPred, to the same dataset, which comprises three subsets: HemoPI-1, HemoPI-2, and HemoPI-3, each with distinct features and characteristics.

- *HemoPI-1 dataset* [8] This dataset includes 552 experimentally verified hemolytic peptides from the Hemolytik database [25] as positive examples and an equal number of randomly generated peptides from the Swiss-Prot database [26] as negative examples. The main and validation datasets were randomly split from this dataset, containing 552 positive and 552 negative examples.
- *HemoPI-2 dataset* [8] Created to distinguish between high and low hemolytic activity peptides, this dataset includes 552 positive examples from the Hemolytik database and 462 negative examples selected based on weak hemolytic activity or failure to meet HemoPI-1 criteria.
- *HemoPI-3 dataset* [8] Comprising 1623 peptides from the DBAASP [27] and Hemolytik databases, this dataset differentiates between highly hemolytic and poorly hemolytic peptides, containing 885 positive and 738 negative examples.
- *RNN-Hem dataset* [16] Developed by Capecchi et al. in 2021, this dataset includes 1359 positive examples from the DBAASP database and 1198 negative examples from Swiss-Prot, representing peptides with and without hemolytic activity, respectively.
- *Hlppredfuse dataset* [24] Created by Hasan et al., this dataset consists of 1066 positive examples from the Hemolytik database and 2422 negative examples from the PEPred-SUITE method [28], representing peptides with and without hemolytic activity, respectively.
- *AMP-Combined dataset* [17] This dataset is a combination of HemoPI-1, RNN-Hem, and Hlppredfuse datasets, including 3007 positive and 4172 negative examples. Created by Salem et al., it was used to evaluate a deep learning-based method for predicting hemolytic activity in peptides.

To conduct a thorough study, we integrated the HemoPI-1, HemoPI-2, HemoPI-3, RNN-Hem, Hlppredfuse, and AMP-Combined datasets into a unified dataset termed the “Integrated Hemolytic Activity Dataset (IHAD).” This dataset initially comprised 7451 positive and 9544 negative examples, sourced from various origins to ensure a rich and heterogeneous representation of hemolytic and non-hemolytic peptides. The diversity within IHAD facilitates a more robust evaluation of the proposed model’s performance across different scenarios, considering variations in data quality, composition, balance, and representation among the individual datasets. To ensure data integrity and prevent bias, duplicate peptides were removed before training, reducing the dataset to 3455 positive and 5566 negative examples. On average, peptides in IHAD have a sequence similarity of 23.3%, which underscores the dataset’s diversity. This diversity enhances the model’s adaptability and generalization, providing a comprehensive understanding of its effectiveness in predicting hemolytic activity in peptides.

**Table 1:** An overview of all datasets

Dataset	Source	Positive Set	Negative Set
HemoPI-1	Chaudhary et al. [8]	552	552
HemoPI-2	Chaudhary et al. [8]	552	462
HemoPI-3	Chaudhary et al. [8]	885	738
RNN-Hem	Capecchi et al. [16]	1359	1198
Hlppredfuse	Hasan et al. [24]	1096	2422
AMP-Combined	Salem et al. [17]	3007	4172
IHAD	–	3455	5566

### Data representation

In deep learning, how we represent data is critical for analyzing peptide sequences. One effective method for representing peptide sequences is one-hot encoding [29]. This technique converts each amino acid into a unique binary vector, where one element is set to 1, and all others are set to 0. This allows machine learning algorithms to process peptide sequences as numerical data, making it easier to detect patterns and relationships within the data.

**Fig. 1:**
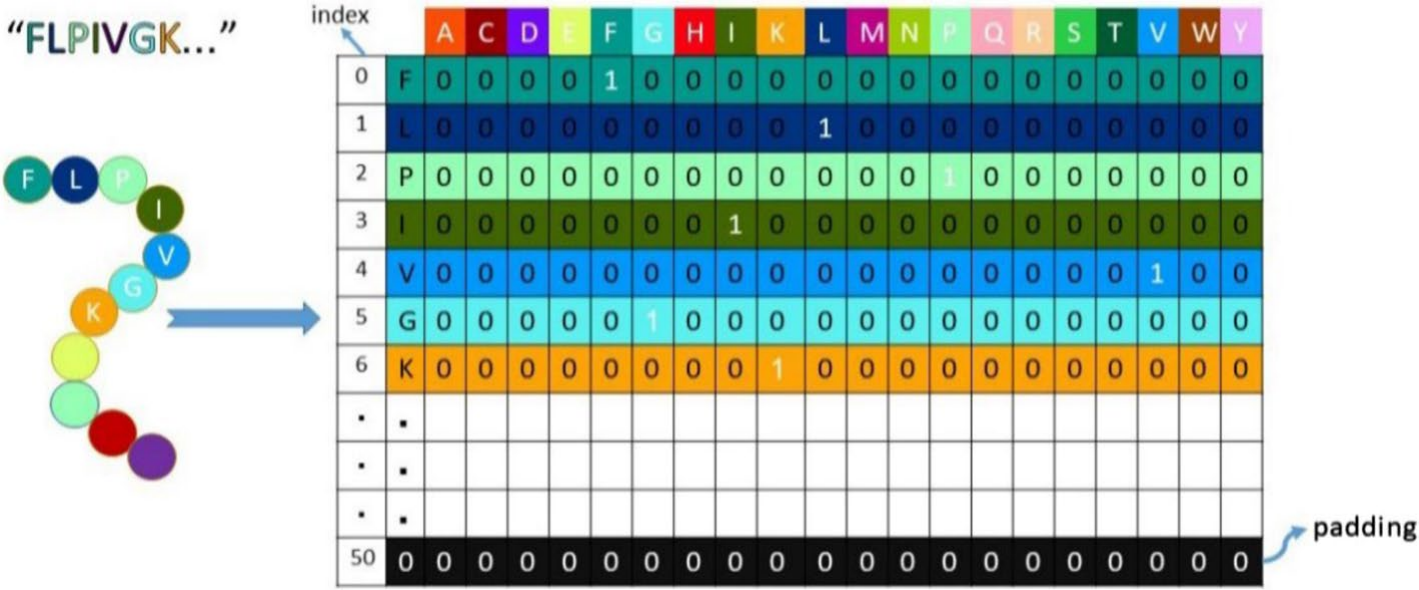
One-Hot encoding applied to the peptide sequence

Using one-hot encoding, each amino acid in a peptide sequence is mapped to a unique binary vector, resulting in a matrix where columns correspond to amino acids and rows represent positions within the sequence. This structured numerical format allows deep learning models to interpret the sequences more effectively. As shown in Fig. 1, each amino acid is assigned a specific binary vector, enhancing the model’s ability to utilize the full informational content of the peptide sequences. This encoding approach ultimately improves the accuracy of hemolytic activity predictions.

**Fig. 2:**
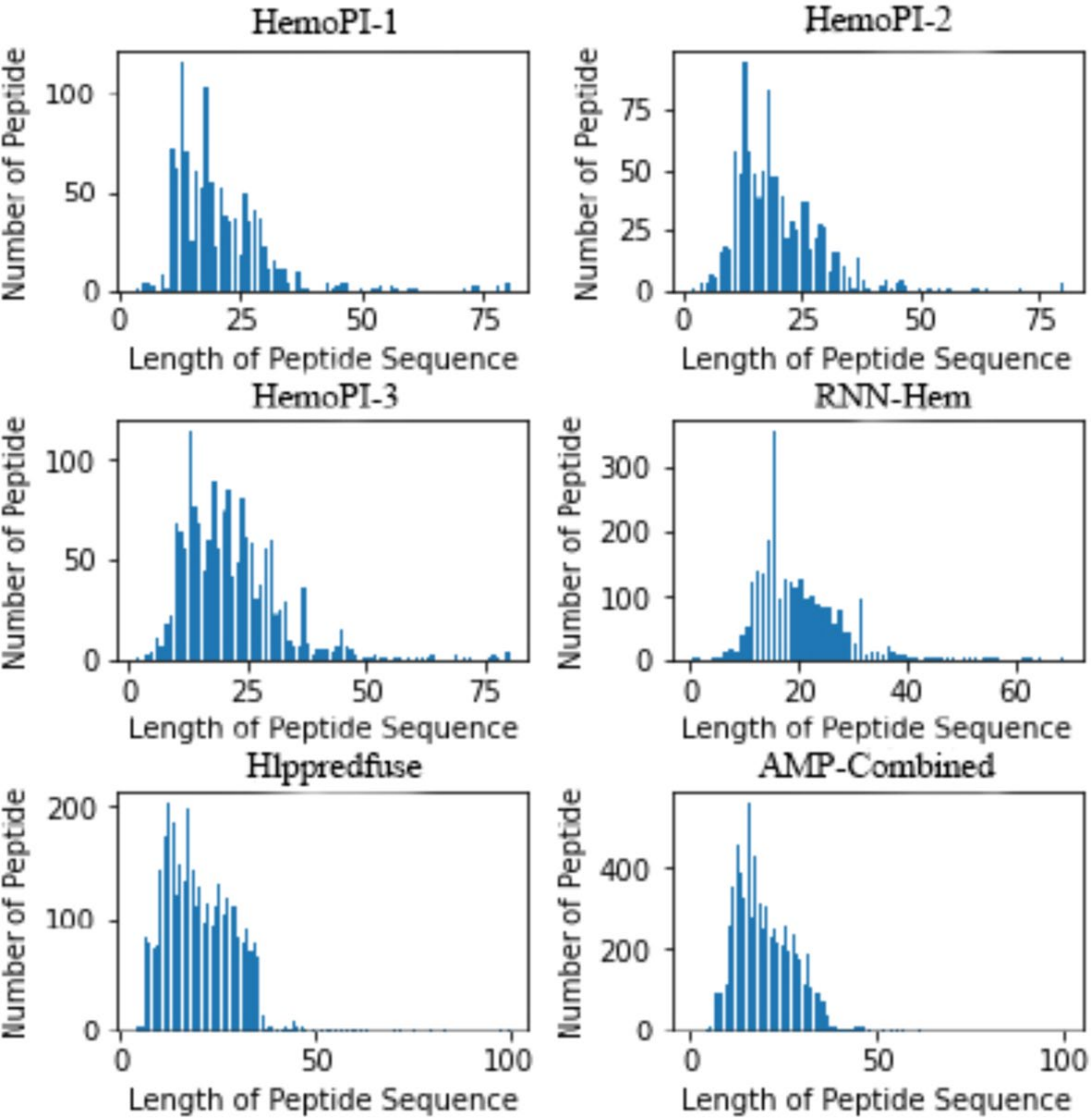
Distribution of peptide lengths in the six datasets

Figure 2 shows the peptide sequence length distribution for the six datasets. A 99% information threshold was used, resulting in a maximum length of 50 amino acid residues for the peptide sequences. Every peptide sequence exceeding or falling below the threshold length was truncated or supplemented, respectively. For example, the peptide sequence:

“MSGIVEAISNAVKSGLDHDWVMGTSIADVVAKGADFIAGF”.

Would be padded with 10 zeros to create a fixed-length sequence of 50 amino acids. Each amino acid in the sequence would then be represented by a unique binary vector of length 20, resulting in a matrix of size 50 × 20.

### Deep neural network for hemolytic activity prediction

The CNN architecture for predicting hemolytic activity comprises several specialized layers, including convolutional, pooling, and dense layers. These layers extract and process local patterns from the input matrix, with convolutional layers utilizing filters to identify important features, pooling layers reducing the dimensionality of the feature maps, and dense layers making the final classification based on the processed data.

Our approach employs a straightforward CNN model architecture. Initially, peptide data undergoes preprocessing, which involves converting peptide sequences into a numerical format understandable by CNN. One common method is one-hot encoding, where each amino acid in the peptide is represented by a vector of zeros, with a 1 at the corresponding index of the amino acid in the sequence. The preprocessed data is then passed through one or more convolutional layers, which extract features by convolving the data with learned filters. Each filter detects specific patterns, such as the presence of hydrophobic or positively charged amino acids.

Following each convolutional layer, a pooling layer is typically used to reduce the data’s dimensionality by summarizing information in small regions of the input, thereby preventing overfitting to the training data. The output from the convolutional and pooling layers is fed into one or more fully connected layers, which learn non-linear relationships between the extracted features and the output classification (hemolytic or non-hemolytic). The model’s final layer, the output layer, consists of one neuron for each possible output class. The neuron with the highest activation indicates the predicted class for the input peptide.

**Fig. 3:**
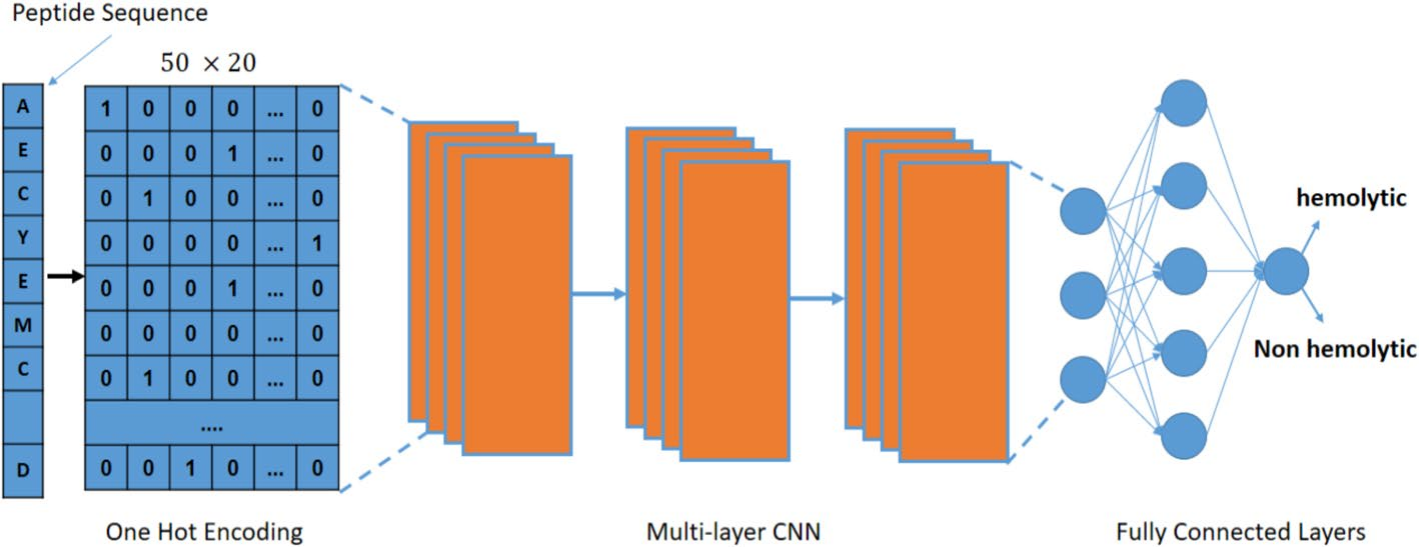
Predicting hemolytic activity of peptides model architecture

As shown in Fig. 3, this architecture enables the CNN to effectively process and classify peptide data, identifying hemolytic activity based on the features extracted through the network’s layers.

In the training process, a set of carefully selected hyperparameters are utilized to maximize the performance of our model. These hyperparameters, which are predetermined before the training begins, have a significant impact on the model’s behavior but are not adjusted during training. Examples of such hyperparameters in this context include the number of filters and their sizes in the convolutional layers, the size of the pooling windows in the pooling layers, the number of neurons in the fully connected layers, the learning rate of the optimizer, the number of training epochs, and the batch size. Refer to Table 2 for a list of the specific hyperparameters utilized in the training process. The model makes use of six convolutional layers with varying numbers of filters to effectively extract a wide range of features from peptide sequences. These features are then condensed into a single dense layer. Incorporating ReLU activation functions allows for non-linear transformations while using a sigmoid function at the output layer produces probability scores. Our approach processes data in batches of 32 and adopts a prudent learning rate of 0.0001 to facilitate the gradual refinement of model parameters. Additionally, utilizing the Adam optimizer enables efficient navigation of the optimization landscape, while the Binary Cross Entropy loss function guides the model toward precise predictions.

**Table 2:** Parameter settings for the proposed model

Parameters	Value
Number of convolutional layers	6
Number of dense layers (FC)	1
Number of filters	[2*32,2*64,2*128]
Filter length	[11,11,7,5,3,3]
Hidden neurons	[32]
Activation function (CNN)	ReLU
Activation function (FC)	ReLU
Activation function output	Sigmoid
Batch size	32
Learning rate	0.0001
Optimizer	Adam
Loss function	Binary cross entropy

### Model evaluation

To evaluate the performance of the CNN model, we used some evaluation metrics. These metrics are namely: accuracy (Acc), precision, recall, and Matthews’s correlation coefficient (MCC). These equations are defined as follows:

1 $$\text{Acc} = \frac{TP+TN}{TP+TN+FP+FN} \times 100$$

2 $$\text{Precision} = \frac{TP}{TP+FP} \times 100$$

3 $$Recall = \frac{TP}{TP+FN} \times 100$$

4 $$\text{Mcc} = \frac{TP \times TN - FP \times FN}{\sqrt{(TP + FP)(TP + FN)(TN + FP)(TN + FN)}}$$

Where TP denotes True Positives, TN represents True Negatives, FP corresponds to False Positives, and FN stands for False Negatives.

## Results and discussion

The proposed CNN model was evaluated on six different datasets using the evaluation metrics outlined in Sect. Model Evaluation, demonstrating high accuracy, precision, recall, and MCC across all datasets. This highlights the model’s robustness in predicting the hemolytic activity of antimicrobial peptides. We used 80% of the dataset for training purposes, and within this training portion, 20% was reserved as the validation set. The remaining 20% of the dataset was used as the test set to evaluate the final performance of the model. The validation set plays a crucial role in monitoring performance and preventing overfitting, with the optimal training epoch determined by the highest accuracy achieved on the validation set. By training and testing the model individually on each dataset, we were able to thoroughly evaluate its performance and generalization across datasets with varying characteristics, ensuring a comprehensive assessment of its predictive capabilities.

In particular, the model performed exceptionally well on the HemoPI-1 dataset, achieving an accuracy of 96.38%, a precision of 95.19%, a recall of 97.06%, and an MCC of 0.9274. Similarly, high performance was observed on the Hlppredfuse dataset, with an accuracy of 94.89%, a precision of 94.66%, a recall of 88.64%, and an MCC of 0.879. The detailed performance metrics for all six datasets are summarized in Table 3.

**Table 3:** Performance of the proposed CNN model on the six datasets

Dataset	Accuracy%	Precision%	Recall%	MCC
HemoPI-1 [8]	96.38	95.19	97.06	0.9274
HemoPI-2 [8]	77.83	74.42	88.88	0.5614
HemoPI-3 [8]	80.31	75.37	82.94	0.6051
RNN-Hem [16]	80.66	83.00	78.95	0.6142
Hlppredfuse [24]	94.89	94.66	88.64	0.8799
AMP-Combined [17]	87.81	79.04	84.39	0.7484
IHAD	90.00	87.49	87.57	0.7918

Table 4 presents a comparative evaluation of the proposed CNN model against the existing HemoPI and HemoPred classifiers on the HemoPI-1, HemoPI-2, and HemoPI-3 datasets. The evaluation metrics used include Accuracy, Precision, Recall, and MCC, which collectively measure the classifiers’ effectiveness in predicting hemoglobin variants.

For the HemoPI-1 dataset, all three classifiers demonstrated high accuracy and recall. Notably, the proposed CNN model achieved accuracy (96.38%) and MCC (0.9274), outperforming the HemoPred classifier. HemoPI achieved the highest accuracy (96.4%) and MCC (0.93), outperforming our proposed CNN model. In the case of the HemoPI-2 dataset, the performance of all classifiers was comparatively lower than that observed for the HemoPI-1 dataset. Accuracy values ranged between 75.7% and 77.83%, recall values between 78.2% and 88.88%, and MCC values between 0.51 and 0.5614. For the HemoPI-2 dataset, the CNN achieved the highest accuracy (77.83%) and MCC (0.5614), although the differences in performance were modest. For the HemoPI-3 dataset, the proposed CNN model again showed promising results, achieving the highest MCC (0.6051) and a recall value of 82.94%. Although the HemoPred classifier achieved the highest recall (85.20%), the accuracy values for all classifiers were similar, hovering around 79%. Precision values for the HemoPI and HemoPred classifiers were not reported. The proposed CNN model delivered robust performance across datasets, with high accuracy, precision, recall, and MCC values, demonstrating its potential as an effective tool for predicting hemoglobin variants.

**Table 4:** Comparison of the proposed CNN model with the previous methods on HemoPI datasets

Dataset	Classifier	Accuracy%	Precision%	Recall%	MCC
HemoPI-1	HemoPI[8]	**96.4**	–	96.4	**0.93**
	HemoPred[9]	96.18	–	95.64	0.92
	Proposed CNN	96.38	**95.19**	**97.06**	0.9274
HemoPI-2	HemoPI[8]	75.7	–	78.2	0.51
	HemoPred[9]	76.82	–	78.91	0.53
	Proposed CNN	**77.83**	**74.42**	**88.88**	**0.5614**
HemoPI-3	HemoPI[8]	77.16	–	81.92	0.54
	HemoPred[9]	79.81	–	**85.20**	0.56
	Proposed CNN	**80.31**	**75.37**	82.94	**0.6051**

Bolded values indicate the best-performing results

Tables 5 and 6 present a comparative analysis of the proposed CNN model against various existing classifiers on the RNN-Hem and HLPpred-Fuse datasets providing a comprehensive assessment of each model’s performance.

On the RNN-Hem dataset (Table 5), the proposed CNN model demonstrated superior performance with an accuracy of 80.66%, precision of 83.00%, recall of 78.95%, and MCC of 0.6142. These results indicate a modest improvement over the SVM-Hem, RF-Hem, RNN-Hem, and AMPDeep classifiers, with the CNN model achieving the highest precision and MCC values. Specifically, the model outperformed SVM-Hem, which recorded an accuracy of 73%, precision of 72%, recall of 58%, and MCC of 0.44, and RF-Hem, which achieved 77% accuracy, 81% precision, 60% recall, and an MCC of 0.53. Additionally, it surpassed the performance of the RNN-Hem classifier, which had 76% accuracy, 70% precision, 76% recall, and an MCC of 0.52, as well as AMPDeep, which achieved 79.97% accuracy, 79.88% precision, 83.28% recall, and an MCC of 0.5972.

**Table 5:** Comparison of the proposed CNN model with the previous methods on the RNN-Hem dataset

Classifier	Accuracy%	Precision%	Recall%	MCC
SVM-Hem[16]	73	72	58	0.44
RF-Hem [16]	77	81	60	0.53
RNN-Hem [16]	76	70	76	0.52
AMPDeep[17]	79.97	79.88	**83.28**	0.5972
Proposed CNN	**80.66**	**83.00**	78.95	**0.6142**

Bolded values indicate the best-performing results

On the HLPpred-Fuse dataset (Table 6), the proposed CNN model achieved an outstanding accuracy of 94.89%, precision of 94.66%, recall of 88.64%, and MCC of 0.8799. This performance outstripped that of other classifiers, including HLPpred-Fuse, HemoPI, HemoPred, and AMPDeep. Notably, the proposed CNN model achieved the highest accuracy and MCC, outperforming the HLPpred-Fuse classifier, which reported only recall (84.5%) and MCC (0.823), and HemoPI, with a recall of 80.4% and MCC of 0.754. Additionally, it surpassed HemoPred’s recall of 65.2% and MCC of 0.34, and AMPDeep, which achieved 93.69% accuracy, 86.67% precision, 88.24% recall, and an MCC of 0.8324.

**Table 6:** Comparison of the proposed CNN model with the previous methods on the HLPpred-Fuse dataset

Classifier	Accuracy%	Precision%	Recall%	MCC
HLPpred-Fuse[24]	–	–	84.5	0.823
HemoPI[8]	–	–	80.4	0.754
HemoPred[9]	–	–	65.2	0.34
AMPDeep[17]	93.69	86.67	88.24	0.8324
Proposed CNN	**94.89**	**94.66**	**88.64**	**0.8799**

Bolded values indicate the best-performing results

Table 7 presents the comparison of the proposed CNN model with AMPDeep on the combined dataset. The results show that the proposed CNN model and AMPDeep have similar accuracy values, but the proposed CNN model outperforms AMPDeep in terms of recall, and MCC. AMPDeep achieved a higher precision of 90.91% than our proposed CNN model. Specifically, the proposed CNN model achieved an accuracy of 87.81%, precision of 79.04%, recall of 84.39%, and MCC of 0.7484, while AMPDeep achieved an accuracy of 86%, precision of 90.91%, recall of 80%, and MCC of 0.7252. These results suggest that the proposed CNN model is a competitive approach for predicting hemolytic activity on the combined dataset.

**Table 7:** Comparison of the proposed CNN model with the previous methods on the AMP-Combined dataset

Classifier	Accuracy%	Precision%	Recall%	MCC
AMPDeep[17]	86	**90.91**	80	0.7252
Proposed CNN	**87.81**	79.04	**84.39**	**0.7484**

Bolded values indicate the best-performing results

Table 8 provides a comparative analysis of the proposed CNN model with AMPDeep on the IHAD dataset. The results indicate that the proposed CNN model consistently outperforms AMPDeep across various performance metrics. While both models exhibit high accuracy values, the proposed CNN model demonstrates superior precision, recall, and Matthews Correlation Coefficient (MCC).

**Table 8:** Comparison of the proposed CNN model with the previous methods on the IHAD dataset

Classifier	Accuracy%	Precision%	Recall%	MCC
AMPDeep[17]	89.37	86.20	87.47	0.7793306
Proposed CNN	**90.00**	**87.49**	**87.57**	**0.7918**

Bolded values indicate the best-performing results

To assess the importance of CNN layers, we conducted two ablation experiments. First, we replaced the CNN with RNN layers (LSTM) to capture long-range dependencies in the peptide sequences. However, as shown in Table 9, the RNN-based model exhibited a slight drop in performance compared to the CNN model, particularly in accuracy and MCC, indicating that CNNs are more effective at extracting local features for this task. This suggests that localized patterns within peptide sequences are more important than capturing the entire sequence context when predicting hemolytic activity. In the second experiment, we removed the CNN layers altogether, passing the one-hot encoded peptide sequences directly to the fully connected layers. As reflected in Table 9, this significantly reduced the model’s performance, with a marked decrease in accuracy and MCC. This result further confirms that CNN layers are crucial for the model’s ability to extract and learn from meaningful features within peptide sequences. Without CNNs, the model struggles to classify hemolytic and non-hemolytic peptides effectively.

**Table 9:** Ablation study results comparison

Dataset	Dataset	Accuracy%	Precision%	Recall%	MCC
Proposed CNN	HemoPI-1[8]	96.38	95.19	97.06	0.9274
	HemoPI-2[8]	77.83	74.42	88.88	0.5614
	HemoPI-3[8]	80.31	75.37	82.94	0.6051
	RNN-Hem[16]	80.66	83.00	78.95	0.6142
	Hlppredfuse[24]	94.89	94.66	88.64	0.8799
	AMP-Combined [17]	87.81	79.04	84.39	0.7484
	IHAD	90.00	87.49	87.57	0.7918
Based on RNN	HemoPI-1[8]	94.12	94.95	92.16	0.8817
	HemoPI-2[8]	70.94	70.59	77.77	0.4147
	HemoPI-3[8]	76.31	71.24	80.00	0.5247
	RNN-Hem[16]	78.91	77.82	83.08	0.5778
	Hlppredfuse[24]	92.76	87.22	90.00	0.8331
	AMP-Combined [17]	87.60	78.19	87.58	0.7473
	IHAD	88.97	87.16	85.00	0.7695
without CNN	HemoPI-1[8]	92.76	93.88	90.20	0.8545
	HemoPI-2[8]	67.98	68.38	74.07	0.3547
	HemoPI-3[8]	73.85	68.95	77.64	0.4752
	RNN-Hem[16]	75.39	76.52	75.94	0.5072
	Hlppredfuse[24]	92.19	88.73	85.91	0.8168
	AMP-Combined [17]	86.28	76.52	83.22	0.7173
	IHAD	84.61	80.89	80.66	0.6795

**Fig. 4:**
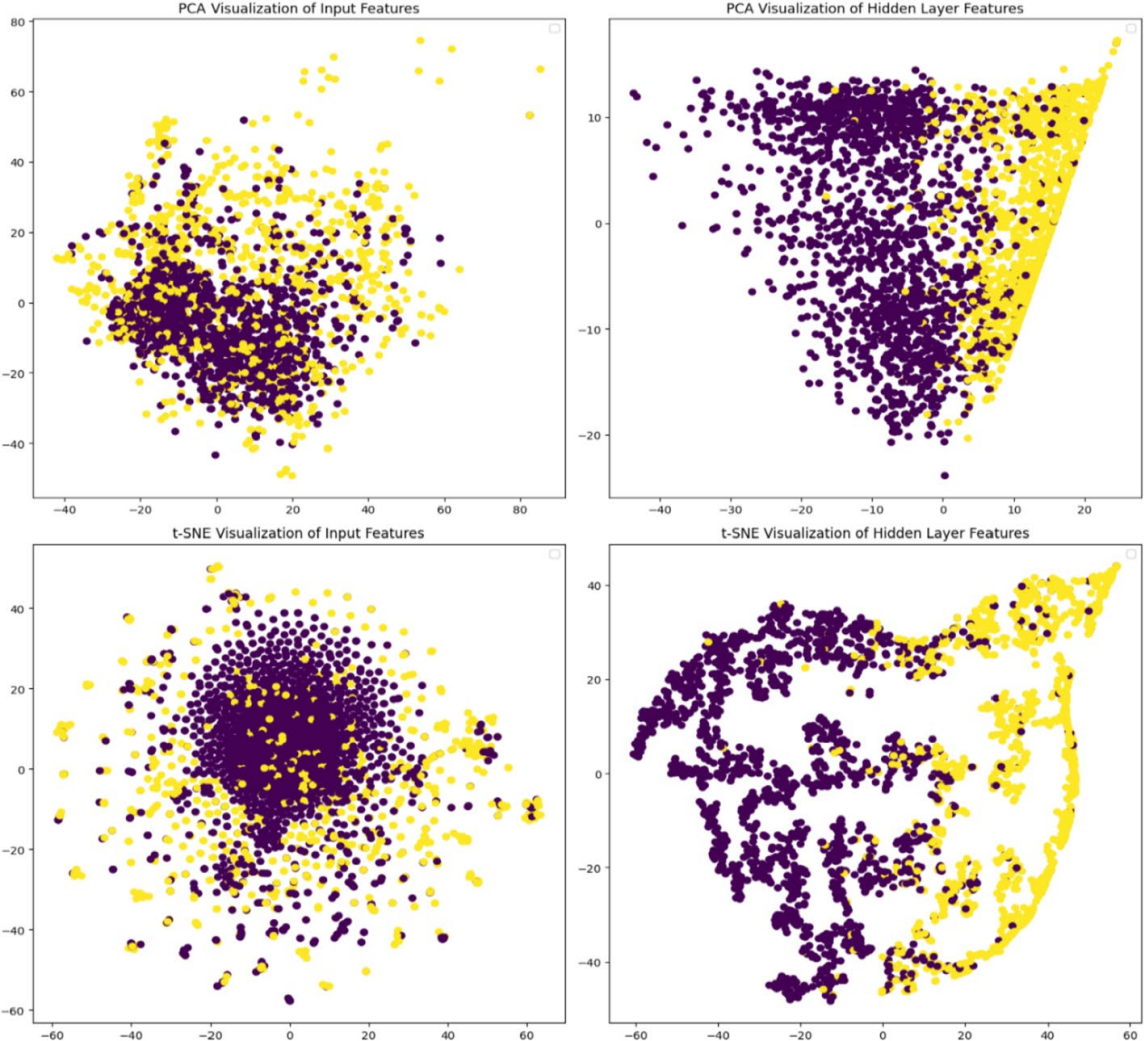
Dimensionality reduction visualizations using PCA (top) and t-SNE (bottom) for both the input features (left) and the hidden layer features (right) of the CNN-based model

To better understand the model’s handling and transformation of input features, we applied dimensionality reduction techniques, including Principal Component Analysis (PCA) and t-SNE, as shown in Fig. 4. The visualizations on the left demonstrate the clustering of input features, while those on the right show how the CNN layers progressively separate the hemolytic and non-hemolytic peptides. The increased separation in the hidden layer representations suggests that the model effectively transforms the input data, enhancing classification performance. The PCA plot shows a well-defined boundary, and the t-SNE analysis further emphasizes the model’s ability to cluster the data more distinctly, indicating CNN’s effectiveness in feature extraction and classification improvement on datasets.

**Fig. 5:**
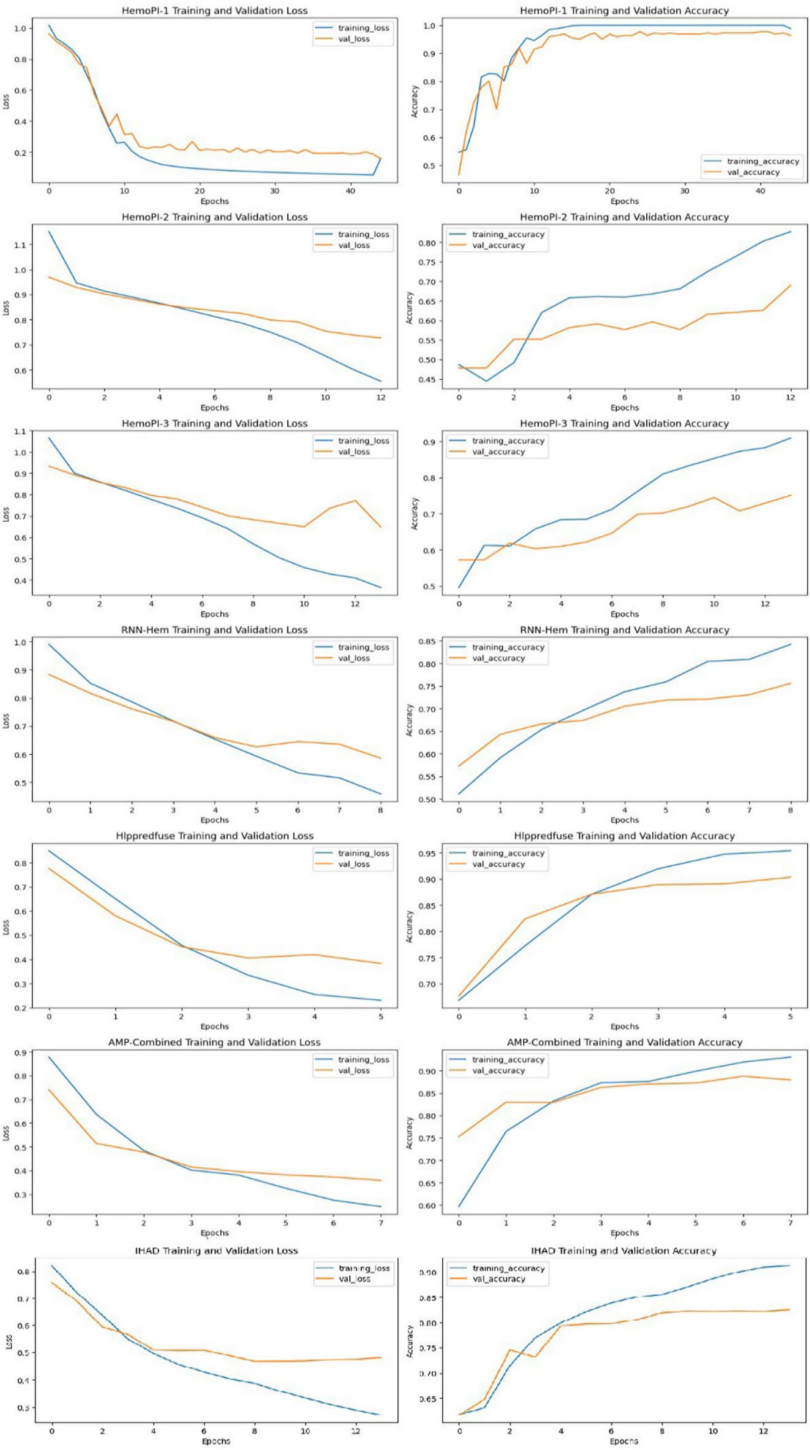
Model accuracy and loss curve for seven datasets

Figure 5 shows the training and validation loss (left column) and training and validation accuracy (right column) curves for the proposed CNN model across six different datasets: HemoPI-1, HemoPI-2, HemoPI-3, RNN-Hem, Hlppredfuse, AMP-Combined, and IHAD. Each subplot presents the model’s performance over multiple epochs, allowing a visual assessment of how well the model generalizes to unseen data. Generally, the training loss decreases and the accuracy increases with the number of epochs, indicating effective learning. However, variations in the validation curves suggest the potential for overfitting in some cases, which can be addressed by tuning hyperparameters or implementing regularization techniques.

## Conclusion

Our research introduces a novel deep neural network model leveraging the power of CNN to accurately predict the hemolytic activity of AMPs. By utilizing one-hot encoding, we represent peptide sequences as 50 × 20 matrices, effectively capturing the sequence information for deep learning. The CNN architecture, comprising multiple convolutional, pooling, and dense layers, extracts essential features and successfully classifies peptides based on their hemolytic activity. Homology statistics indicate dataset diversity, with a minimum similarity of 0%, maximum of 67.3%, average of 23.3%, and standard deviation of 9.2%, ensuring the model was evaluated on a broad range of peptide sequences. Extensive experiments conducted on six diverse datasets demonstrate the exceptional performance of our approach across various metrics, including accuracy, precision, recall, and MCC. Compared to existing state-of-the-art predictors, our CNN model consistently achieves superior results, highlighting its robustness and effectiveness. The remarkable predictive capability of our proposed model positions it as a valuable tool in the design and development of new antimicrobial peptides with reduced hemolytic activity. This advancement is crucial for enhancing the clinical utility of AMPs in treating bacterial infections, as it addresses the challenge of hemolysis, which has previously limited their therapeutic application. Future work will consider incorporating physicochemical properties, such as hydrophobicity, charge, and molecular weight, during feature selection to provide additional insights into peptide behavior and potentially improve the model’s predictive power. Future research may focus on fine-tuning hyperparameters and exploring more sophisticated CNN architectures to further enhance model performance. Additionally, integrating structural data and evaluating the model’s applicability to novel datasets and various types of AMPs could provide deeper insights and improve its generalization capabilities. Further studies are essential to confirm these findings and expand the model’s applicability in practical, clinical settings.

## Summary Points

1. AMPs combat microorganisms widely but risk hemolysis, limiting clinical utility.
2. Computational approaches, like CNN-based models, predict AMPs’ hemolytic potential.
3. One-hot encoding represents peptide sequences in the CNN architecture.
4. The model, trained on various datasets, outperforms prior methods, aiding in safer AMP development.

## Data Availability

The data and the scripts for this work are available through GitHub at https://github.com/mohamedelhakim/Hemolytic-End-to-End-Architecture.
